# Co-Occurrence of Leading Lifestyle-Related Chronic Conditions Among Adults in the United States, 2002-2009

**DOI:** 10.5888/pcd10.120316

**Published:** 2013-04-25

**Authors:** Earl S. Ford, Janet B. Croft, Samuel F. Posner, Richard A. Goodman, Wayne H. Giles

**Affiliations:** Author Affiliations: Janet B. Croft, Samuel F. Posner, Wayne H. Giles, Centers for Disease Control and Prevention, Atlanta, Georgia; Richard A. Goodman, Emory University School of Medicine and Centers for Disease Control and Prevention, Atlanta, Georgia, and US Department of Health and Human Services, Washington, DC.

## Abstract

**Introduction:**

Public health and clinical strategies for meeting the emerging challenges of multiple chronic conditions must address the high prevalence of lifestyle-related causes. Our objective was to assess prevalence and trends in the chronic conditions that are leading causes of disease and death among adults in the United States that are amenable to preventive lifestyle interventions.

**Methods:**

We used self-reported data from 196,240 adults aged 25 years or older who participated in the National Health Interview Surveys from 2002 to 2009. We included data on cardiovascular disease (coronary heart disease, angina pectoris, heart attack, and stroke), cancer, chronic obstructive pulmonary disease (emphysema and chronic bronchitis), diabetes, and arthritis.

**Results:**

In 2002, an unadjusted 63.6% of participants did not have any of the 5 chronic conditions we assessed; 23.9% had 1, 9.0% had 2, 2.9% had 3, and 0.7% had 4 or 5. By 2009, the distribution of co-occurrence of the 5 chronic conditions had shifted subtly but significantly. From 2002 to 2009, the age-adjusted percentage with 2 or more chronic conditions increased from 12.7% to 14.7% (*P* < .001), and the number of adults with 2 or more conditions increased from approximately 23.4 million to 30.9 million.

**Conclusion:**

The prevalence of having 1 or more or 2 or more of the leading lifestyle-related chronic conditions increased steadily from 2002 to 2009. If these increases continue, particularly among younger adults, managing patients with multiple chronic conditions in the aging population will continue to challenge public health and clinical practice.

## Introduction

More than 70.0% of deaths in the United States and about 75.0% of health care spending costs are attributable to chronic diseases (1). The 5 leading causes of death — heart disease, cancer, chronic lower respiratory disease, cerebrovascular disease, and diabetes — accounted for more than half of all deaths in 2009 and represent a high percentage of the nation’s health care costs. Other chronic conditions exact a heavy toll in terms of disease, disability, quality of life, and economic costs (1).

Although much is known about the descriptive epidemiology of these 5 leading causes of disease and death, less is known about how commonly these conditions occur together (2–6). Such data could inform clinicians, public health professionals, policy makers, and health insurers. In recognition of the challenges to public health and the health care system posed by people with multiple chronic conditions, the US Department of Health and Human Services (HHS) developed a strategic framework for multiple chronic conditions (7,8). This framework outlines goals, objectives, and strategies to address clinical and public health system changes to improve health: maximize use of self-care and related services; provide information to public health, social services, and clinical care providers; and promote research to address gaps in knowledge.

Because the roots of the chronic conditions that are the leading causes of morbidity and mortality can be traced to lifestyle factors — principally smoking, diet, and physical activity — it is likely that, despite significant reductions in the prevalence of smoking, the continuing erosion of a low-risk lifestyle profile (9) could result in an increase in the incidence, prevalence, and co-occurrence of lifestyle-related chronic conditions. In response to the HHS strategic framework’s recommendations for research to address the epidemiology of multiple chronic conditions (7,8), our study’s main objectives were to assess the prevalence of co-occurrence of leading lifestyle-related chronic conditions and to examine trends in the prevalence of these conditions from 2002 to 2009.

## Methods

We used data from the National Health Interview Survey (NHIS) for the years 2002 through 2009 (10). For each year, NHIS used a complex sampling design to select a sample representing the noninstitutionalized civilian population in the United States. Interviewers visited participants in their homes and conducted computer-assisted personal interviews. The response rates for participants in the sample adult component of the NHIS in consecutive years from 2002 through 2009 were: 2002, 74.3%; 2003, 74.2%; 2004, 72.5%; 2005, 69.0%; 2006, 70.8%; 2007, 67.8%; 2008, 62.6%; and 2009, 65.4%. Because this study involved the use of publicly available data sets, approval by an institutional review board was not required.

We included the following diagnosed conditions in our analyses: coronary heart disease, angina pectoris, heart attack, stroke, cancer, emphysema, chronic bronchitis, diabetes, and arthritis. These chronic conditions were selected on the basis of the following considerations: 1) these conditions are among the leading causes of death and disability, and 2) modifiable lifestyle factors are important determinants of these conditions. We used a set of NHIS questions to identify participants with chronic conditions (Appendix). Although the question about arthritic conditions includes some forms of arthritis that are unlikely to be heavily influenced by lifestyle-related behaviors, the predominant constituent of this disease is likely to be osteoarthritis, which does have a strong lifestyle-related component. We combined coronary heart disease, angina pectoris, heart attack, and stroke into a single category of cardiovascular disease. We also combined emphysema and chronic bronchitis into a single category of chronic obstructive pulmonary disease. For the question about diabetes, we considered participants who responded that they had borderline diabetes as not having diabetes. Participants who responded “do not know” to any question were classified as not having that condition. The analyses began with 2002 data because the question about arthritis was first included in the NHIS in that year.

Covariates were age, sex, race/ethnicity (non-Hispanic white, non-Hispanic black, Hispanic, and non-Hispanic other), and educational status (did not graduate from high school, high school graduate or equivalent, education beyond high school). These covariates were selected because the prevalence of chronic conditions shows a strong age-related gradient and differs by sex, race/ethnicity, and educational status.

Analyses were restricted to adults aged 25 years or older because educational attainment is more stable in this age group than in younger age groups. Additionally, the prevalence of the selected conditions is lower among people under age 25. Age adjustment was performed by using the direct method and the distribution of 3 age groups from the projected year 2000 population (25–44 y, 45–64 y, and ≥65 y). Comparisons of estimates were made by using *t* tests for dichotomous variables and by using χ^2^ tests for categorical variables with more than 2 levels. Estimates of the number of adults aged 25 years or older with 1 or more chronic conditions were calculated by multiplying the unadjusted proportion by intercensal estimates of the resident population of the United States. Tests for trend were conducted by using orthogonal linear contrasts and by using log-binomial regression models that included age, sex, race/ethnicity, and educational status as covariates. Data management was performed in SAS 9.3 (SAS Institute Inc, Cary, NC), and prevalence estimates and *P* values were calculated by using SUDAAN version 11.0.0 (Research Triangle Institute, Research Triangle Park, North Carolina). Sampling weights were used to produce estimates and conduct statistical tests.

## Results

From a total of 198,710 participants aged 25 years or older in the 8 years (2002–2009), we included 196,240 participants in the analyses, after excluding participants with missing values for education (n = 2,290), cancer (n = 110), diabetes (n = 90), chronic obstructive pulmonary disease (n = 142), and arthritis (n = 138). During the period studied, mean age increased from 48.8 years to 49.8 years (*P* < .001); the percentage of non-Hispanic whites decreased from 74.5% to 70.0% (*P* < .001), and the percentage of participants who had completed high school or its equivalent increased from 83.9% to 85.6% (*P* < .001). The sex distribution remained relatively stable.

Of the 5 chronic conditions, the age-adjusted prevalence of cancer, diabetes, and arthritis increased significantly from 2002 to 2009 (Table 1). The prevalence of cardiovascular disease changed little in that same period, especially between 2004 and 2009. The prevalence of chronic obstructive pulmonary disease remained unchanged. The prevalence of having 1 or more, 2 or more, and 3 or more conditions increased significantly.

**Table 1:** Age-Adjusted Percentages (Standard Error) of Adults Aged ≥25 Years Who Report Having ≥1, ≥2, or ≥3 Leading Lifestyle-Related Chronic Conditions or Individual Conditions and Who Have ≥2 Self-Reported Leading Lifestyle-Related Chronic Conditions^a^ by Selected Sociodemographic Characteristics, by Year, National Health Interview Survey, 2002-2009

Variable	2002 (N = 27,279)	2003 (N = 27,168)	2004 (N =27665)	2005 (N = 27,961)	2006 (N = 21,274)	2007 (N = 20,636)	2008 (N = 19,502)	2009 (N = 24,755)	P Value^b^

	% (SE)
**Chronic disease**									
Cardiovascular disease	8.7 (0.2)	8.4 (0.2)	9.0 (0.2)	9.1 (0.2)	8.9 (0.2)	8.7 (0.2)	9.1 (0.2)	9.1 (0.2)	.075
Cancer	8.1 (0.2)	7.5 (0.2)	8.0 (0.2)	8.4 (0.2)	8.0 (0.2)	8.2 (0.2)	8.8 (0.2)	9.0 (0.2)	<0.001
Diabetes	7.4 (0.2)	7.4 (0.2)	7.9 (0.2)	8.3 (0.2)	8.6 (0.2)	8.6 (0.2)	9.0 (0.2)	9.7 (0.2)	<.001
Chronic obstructive pulmonary disease	5.9 (0.2)	5.3 (0.2)	5.8 (0.2)	5.6 (0.2)	5.9 (0.2)	4.9 (0.2)	5.6 (0.2)	6.0 (0.2)	.990
Arthritis	23.5 (0.3)	24.2 (0.3)	24.4 (0.3)	24.0 (0.3)	23.5 (0.3)	23.1 (0.4)	24.9 (0.3)	25.1 (0.4)	.021
≥1 Condition	36.5 (0.3)	36.1 (0.3)	37.3 (0.3)	37.5 (0.3)	36.6 (0.4)	36.3 (0.4)	38.1 (0.4)	38.7 (0.4)	<.001
≥2 Conditions	12.7 (0.2)	12.4 (0.2)	13.2 (0.2)	13.2 (0.2)	13.3 (0.3)	12.8 (0.3)	14.2 (0.3)	14.7 (0.3)	<.001
≥3 Conditions	3.6 (0.1)	3.6 (0.1)	3.8 (0.1)	3.9 (0.1)	4.2 (0.2)	3.7 (0.2)	4.2 (0.1)	4.5 (0.2)	<.001
**Sociodemographic characteristics**									
**Age, y**									
25–44	2.5 (0.2)	2.2 (0.1)	2.8 (0.2)	2.8 (0.2)	2.6 (0.2)	2.5 (0.2)	3.1 (0.3)	3.2 (0.2)	.003
45–64	13.9 (0.5)	13.6 (0.4)	13.6 (0.4)	13.9 (0.4)	14.1 (0.5)	13.3 (0.5)	15.4 (0.5)	16.1 (0.5)	<.001
≥65	34.6 (0.7)	34.6 (0.7)	36.9 (0.7)	36.4 (0.7)	37.2 (0.9)	36.1 (0.9)	38.4 (0.9)	39.6 (0.8)	<.001
**Sex^c^**									
Men	12.4 (0.3)	12.0 (0.3)	13.1 (0.3)	12.4 (0.3)	12.8 (0.4)	12.3 (0.4)	12.9 (0.4)	14.7 (0.4)	<.001
Women	13.1 (0.3)	12.8 (0.3)	13.5 (0.3)	13.9 (0.3)	13.9 (0.4)	13.3 (0.3)	15.4 (0.4)	14.9 (0.4)	<.001
**Race/ethnicity^c^**									
Non-Hispanic white	13.0 (0.2)	13.0 (0.2)	13.6 (0.3)	13.6 (0.2)	13.9 (0.3)	13.2 (0.3)	14.9 (0.4)	15.5 (0.3)	<.001
Non-Hispanic black	13.8 (0.6)	12.6 (0.6)	13.0 (0.6)	13.7 (0.7)	14.3 (0.7)	14.1 (0.6)	14.2 (0.7)	16.0 (0.7)	.002
Hispanic	9.6 (0.7)	8.4 (0.5)	11.2 (0.7)	10.9 (0.6)	10.5 (0.7)	10.4 (0.6)	11.8 (0.6)	11.3 (0.7)	.002
Non-Hispanic other	8.5 (1.2)	8.5 (1.1)	10.2 (1.1)	8.1 (1.0)	8.1 (0.9)	8.8 (1.0)	9.5 (1.0)	7.8 (0.8)	.739
**Education^c^**									
Less than high school graduate or equivalent	16.6 (0.6)	15.1 (0.5)	17.3 (0.6)	17.0 (0.6)	17.1 (0.7)	17.8 (0.7)	18.5 (0.8)	18.9 (0.8)	<.001
High school graduate or equivalent	13.2 (0.4)	12.4 (0.4)	13.4 (0.4)	13.4 (0.4)	13.5 (0.5)	12.8 (0.5)	15.7 (0.5)	16.1 (0.5)	<.001
>High school	11.3 (0.3)	11.6 (0.3)	11.9 (0.3)	11.9 (0.3)	12.0 (0.3)	11.2 (0.4)	12.2 (0.4)	12.9 (0.4)	.006

a Chronic conditions include cardiovascular disease (coronary heart disease, myocardial infarction, angina, or stroke), diabetes, cancer, chronic obstructive pulmonary disease, and arthritis.

b *P* values for linear trend were calculated by using orthogonal polynomial contrasts.

c Adjusted for age.

In 2002, an unadjusted 63.6% of participants did not have any of the 5 leading chronic conditions; 23.9% had 1, 9.0% had 2, 2.9% had 3, and 0.7% had 4 or more (Table 2). By 2009, the distribution of the number of chronic conditions had shifted subtly but significantly for the total study population (*P* < .001) and in the 2 oldest age groups. Although significant, the absolute change was small. The age-adjusted percentage of participants who had at least 2 chronic conditions increased significantly, from 12.7% in 2002 to 14.7% in 2009 (*P* < .001) (Table 1). After adjusting for age, sex, race/ethnicity, and educational status, the increase remained significant (prevalence ratio per year, 1.03; 95% confidence interval, 1.02–1.03).The prevalence increased in all other sociodemographic groups except among Non-Hispanic other participants. The largest relative increases occurred among participants aged 25 to 44 years and those who had graduated from high school or received an equivalent degree.

**Table 2:** Unadjusted Distribution of Co-Occurrence of 5 Major Lifestyle-Related Chronic Conditions Among US Adults Aged 25 Years or Older in 2002 and 2009, by Age Groups, National Health Interview Survey (10)

Age, y	No. of Chronic Conditions	2002, %	2009, %	*P* Value^a^
≥25	0	63.6	59.9	<.001
	1	23.9	24.0	
	2	9.0	10.7	
	3	2.9	3.7	
	4 or 5	0.7	0.9	

25-44	0	83.6	82.8	.187
	1	13.9	14.1	
	2	2.1	2.6	
	3	0.4	0.5	
	4 or 5	0.1	0.0	

45-64	0	56.4	52.7	.001
	1	29.7	31.2	
	2	10.4	12.0	
	3	2.8	3.3	
	4 or 5	0.6	0.8	

≥65	0	28.2	25.5	<.001
	1	37.2	34.9	
	2	23.0	25.4	
	3	9.3	11.2	
	4 or 5	2.3	3.0	

a *P* value from χ^2^ test.

 The unadjusted percentage of participants who had at least 1 chronic condition increased from 36.4% in 2002 to 40.1% in 2009. The estimated number of US adults with 1 or more self-reported chronic conditions increased from approximately 67.9 million in 2002 to 81.3 million in 2009 (Figure). Of the 13.4 million increase, about 6.9 million was due to the increase in prevalence and 6.4 million to population growth. Furthermore, the number with 2 or more self-reported chronic conditions increased from approximately 23.4 million in 2002 to 30.9 million in 2009, and the number with 3 or more self-reported chronic conditions increased from approximately 6.7 million in 2002 to 9.3 million in 2009.

**Figure:**
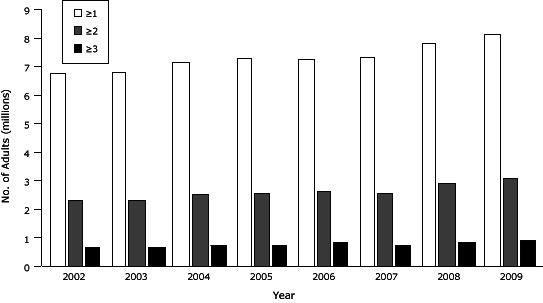
Estimated numbers of US adults aged 25 years or older with self-reported, co-occurring major lifestyle-related chronic conditions, National Health Interview Survey, 2002-2009 (10). **Table:** 

Year	No. of Chronic Conditions		
	≥1	≥2	≥3
2002	67,932,675	23,384,265	6,674,717
2003	68,169,830	23,224,717	6,641,934
2004	71,616,034	25,147,359	7,202,882
2005	73,134,684	25,482,334	7,428,100
2006	72,937,179	26,353,368	8,300,968
2007	73,535,960	25,644,683	7,375,794
2008	78,558,208	29,308,156	8,644,561
2009	81,310,944	30,932,573	9,282,892

The 5 leading chronic conditions form 32 possible mutually exclusive combinations. After age-adjustment, the most common single condition was arthritis (13.3% in 2002 and 13.0% in 2009). The most common combinations of 2 conditions were cancer and arthritis (2.1% in 2002 and 2.2% in 2009), cardiovascular disease and arthritis (2.0% in 2002 and 2.2% in 2009), and diabetes and arthritis (1.5% in 2002 and 2.2% in 2009). The most common combination of 3 conditions was cardiovascular disease, diabetes, and arthritis (0.9% in 2002 and 1.0% in 2009).

Large percentages of adults who had 1 of the 5 chronic conditions also reported having 1 or more additional chronic conditions (Table 3). For participants who reported having cardiovascular disease, chronic obstructive pulmonary disease, or arthritis, the age-adjusted percentage of adults reporting at least 1 additional chronic condition increased significantly from 2002 to 2009 (Table 3). For participants who reported having either cancer or diabetes with at least 1 additional condition, no such trend was evident.

**Table 3:** Age-Adjusted Percentages (Standard Error) of Having One or More Additional Self-Reported Chronic Conditions^a^ Among Adults Aged ≥25 Years Who Have a Chronic Condition, by Year, National Health Interview Survey, 2002-2009

Year	Cardiovascular Disease	Chronic Obstructive Pulmonary Disease	Cancer	Diabetes	Arthritis

	% (SE)
2002	56.6 (2.1)	54.0 (1.4)	47.6 (1.6)	54.1 (1.8)	35.0 (0.8)
2003	58.4 (2.2)	58.4 (1.5)	45.1 (1.6)	49.9 (1.6)	33.1 (0.7)
2004	60.3 (1.8)	58.6 (1.5)	47.7 (1.7)	50.9 (1.6)	35.4 (0.8)
2005	61.0 (2.1)	57.7 (1.5)	47.8 (1.7)	49.7 (1.6)	36.0 (0.8)
2006	61.9 (2.5)	56.6 (1.9)	48.7 (1.9)	48.3 (1.6)	37.4 (1.1)
2007	58.9 (2.4)	60.7 (2.1)	44.3 (2.2)	48.2 (2.0)	35.4 (1.0)
2008	64.9 (2.4)	58.2 (1.9)	48.2 (1.9)	55.9 (2.0)	37.9 (1.1)
2009	68.2 (2.3)	58.5 (1.9)	46.7 (1.9)	52.5 (2.0)	39.2 (0.9)
*P* value^b^	<.001	.005	.782	.591	<.001

a Chronic conditions include cardiovascular disease (coronary heart disease, myocardial infarction, angina, stroke, or hypertension), diabetes, cancer, chronic obstructive pulmonary disease, and arthritis.

b *P* values for linear trend were calculated by using orthogonal polynomial contrasts.

## Discussion

Unhealthy lifestyle behaviors are responsible for much of the disease and death from the leading chronic diseases (11,12). Our analysis of recent national data, which shows that about 1 in 7 adults had at least 2 of 5 chronic conditions associated with disease and death, helps to address a gap in the existing knowledge base. On the basis of our analysis, an estimated 30.9 million adults had 2 or more major lifestyle behavior-related chronic conditions in 2009. Furthermore, the prevalence of having at least 2 such chronic conditions increased by an average 0.26% per year from 2002 to 2009 (*P* = .011), with significant increases in cancer (*P* < .001), diabetes (*P* < .001), and arthritis (*P* = .021) likely contributing to the increase.

Because certain lifestyle behaviors are risk factors for many chronic conditions, including the 5 leading chronic diseases included in this study, improving the behavioral risk factor profile of the population could lower the prevalence of these conditions and decrease their co-occurrence. For example, enormous progress has been made in reducing the prevalence of smoking in the United States (13), and the stable prevalence estimates for chronic obstructive pulmonary disease are likely a reflection of this. However, chronic obstructive pulmonary disease remains the only major chronic disease that has not experienced a large decline in mortality since 1999 (14).

The heavy toll exacted by the co-occurrence of multiple chronic conditions is demonstrated by its effect on death, quality of life, hospitalizations, outpatient visits, health care costs, and other health care metrics (2,4–6,15–17). We found that the largest relative increase in the percentage of adults with 2 or more chronic conditions occurred in the youngest group, albeit over a small baseline. If sustained, this increase would have implications for the health of the nation in future decades.

Not only does the number of chronic conditions have serious implications for disease, death, and health care costs, but specific combinations of chronic conditions may also negatively or positively influence health and economic outcomes. Specific combinations of chronic conditions may affect quality of life (18,19), functional recovery (20), disability (21), health care use (22), health care costs (17,23), and polypharmacy (the use of multiple medications by a patient) (24). Furthermore, combinations of comorbidity may also affect survival after serious conditions such as heart failure (25). For example, the combination of chronic kidney disease and dementia was associated with greatly reduced survival among hospitalized patients with heart failure. A previous analysis of NHIS data that included 9 chronic conditions found that the combinations of hypertension and diabetes, hypertension and heart disease, and hypertension and cancer were the most common 2-condition combinations (26). An analysis of German insurance claims data showed that the most common combination of 3 conditions among 46 chronic conditions included in the study was hypertension, lipid disorder, and chronic low back pain (27). In comparison, we found that the combination of cardiovascular disease and arthritis was the most common 2-condition combination, and the combination of cardiovascular disease, diabetes, and arthritis was the most common 3-condition combination.

Our study has limitations. First, the self-reported nature of the data likely led to an underestimate of the true prevalence of the chronic conditions. For example, the prevalence of self-reported diabetes underestimates the gold standard prevalence estimated from self-reported data and blood measurements of glucose by about a third to a half (28). Recent national data about the trends of cardiovascular disease, cancer, chronic obstructive pulmonary disease, and arthritis based on information other than self-report are not available. Therefore, our results require confirmation with other data based on more rigorous assessments of chronic conditions. Second, we were not able to measure undiagnosed disease; therefore, an alternative explanation of the increase in the percentage of adults having 1 or more chronic conditions is that awareness of these conditions may have improved in the face of a stable prevalence of conditions, thus contributing to the apparent trend. However, the increase in the prevalence of diabetes noted in our study is consistent with data from the National Health and Nutrition Examination Survey in which questionnaires were complemented with measurements of plasma glucose (28).

Another possible limitation is that the decrease in response rates during the study period raises the possibility that the results may have been subject to a bias. If participants who increasingly refused to participate were healthier than participants who opted to participate, a trend showing an increase in multiple chronic conditions may have represented an artifact. However, the lack of information about the health of adults who refused to participate precludes a thorough exploration of this possibility.

The reports of other investigators continue to shape and strengthen our knowledge base characterizing the prevalence and heterogeneity of multiple chronic conditions. Various studies provide estimates of the prevalence of multiple chronic conditions (Table 4). A recent NHIS analysis of data on 9 chronic conditions showed that 21.0% of adults aged 45 to 64 years and 45.3% of adults aged 65 years or older had 2 or more chronic conditions (26). That study examined only adults aged 45 or older. In comparison, we found that 14.7% of adults had 2 or more lifestyle-related chronic conditions in 2009, and 4.5% had 3 or more. Many of these analyses used different sets of chronic conditions in establishing their indices. Prevalence estimates of multiple chronic conditions are clearly influenced by the number of conditions that are considered: the more conditions that are included in a study, the higher the estimates will be. Thus, because we restricted our analyses to 5 chronic conditions that are leading sources of disease and death and that are strongly related to lifestyle factors, the estimates of the noninstitutionalized US population generated in our study are lower than those found elsewhere. Consequently, our analyses yield a complementary perspective on a subset of multiple chronic conditions that had not been previously considered.

**Table 4:** Studies Estimating Prevalence of Chronic Conditions in the United States

Study	Data Source	Ages	Conditions Examined	No. of Chronic Conditions	Prevalence (%)
Fryback et al 1993 (30)	Beaver Dam Health Outcomes Study, 1991-92	45–89 y	28 conditions	0	18.3
				1	20.2
				2	20.2
				3	16.8
				4	11.7
				≥5	12.8

Hwang et al 2001 (31)	Medical Expenditure Panel Survey 1996	All ages	2 physician panels reviewed ICD-9 codes: 111 ICD-9 codes in children, 177 ICD-9 codes in adults, 259 clinical classification system categories	0	59.3
				1	23.7
				2	9.6
				≥3	7.4

Wolff et al 2002 (2)	Medicare 1999	≥65 y	Reviewed ambulatory diagnostic groups to identify ICD-9-CM codes	0	18.0
				1	17.3
				2	21.8
				3	18.8
				≥4	24.1

Anderson and Horvath 2004 (3)	Medical Expenditure Panel Survey 1998	All ages	2 physician panels reviewed ICD-9 codes	**Men**	
				1	22.0
				2	9.0
				3	4.0
				4	2.0
				≥5	2.0
				**Women**	
				1	23.0
				2	12.0
				3	7.0
				4	3.0
				≥5	3.0

Partnership for Solutions 2004 (32)	Medical Expenditure Panel Survey 2001	All ages	2 physician panels reviewed ICD-9 codes	**Men**	
				1	24.0
				2	10.0
				3	5.0
				4	3.0
				≥5	2.0
				**Women**	
				1	24.0
				2	12.0
				3	8.0
				4	4.0
				≥5	4.0

Schneider et al 2009 (5)	Medicare fee-for service 2005	<65–>85 y	21 chronic conditions	0	50.7
				1	29.0
				2	12.7
				≥3	7.6

Paez et al 2009 (6)	Medical Expenditure Panel Survey 2005	All ages	A physician panel reviewed ICD-9 codes: 111 ICD-9 codes in children, 177 ICD-9 codes in adults	0	56.3
				1	19.7
				2	10.7
				≥3	13.3

Anderson 2010 (33)	Medical Expenditure Panel Survey 2006	All ages	2 physician panels reviewed ICD-9 codes	1	22.3
				2	11.8
				3	7.1
				4	3.9
				≥5	4.8

Centers for Medicare and Medicaid Services 2011 (17)	Medicare fee-for-service 2008	<65–>85 y	15 chronic conditions	0-1	33.0
				2-3	33.0
				4	13.0
				5	9.0
				≥6	12.0

Our results provide a new dimension in understanding the increasing burden of chronic diseases in the United States. An increasing percentage of adults are living with 2 or more chronic conditions, and more young people are reporting multiple chronic conditions. These trends, if unabated, could increase the nation’s future health care costs and required health care resources. In particular, several researchers report that increases in the rate of hospitalizations and medical expenditures are related to increases in the number of co-occurring chronic conditions (2,5,6).

The high, increasing prevalence of lifestyle-related multiple chronic conditions provides yet another reason to aggressively promote population-based actions to improve lifestyle behaviors. In many parts of the country, efforts are under way to implement systems and environmental change in schools, communities, and workplaces. A prominent example of such efforts is the Community Transformation Grants program that seeks to build healthier communities and lifestyles through evidence-based approaches to reduce chronic diseases (29).

Although clinicians routinely manage patients who have more than 1 chronic condition, the growing prevalence of patients with multiple chronic conditions may pose additional challenges. First, the large numbers of prescriptions that may be required by such patients may affect a patient’s adherence to taking medications. Second, the risk for adverse reactions from possible interactions among medications increases as the numbers of medications that patients are required to take increases. Finally, the presence of comorbidities may limit the clinician’s therapeutic options. Thus, the coordination of care in such patients poses a serious clinical challenge.

Additional multifaceted research concerning the epidemiology of lifestyle-related multiple chronic conditions is needed to build a more complete understanding of this area. First, studies using large administrative databases would allow a fuller accounting of lifestyle-related conditions and provide sufficient power to characterize the prevalence of unique combinations of conditions. Second, determinants of lifestyle-related chronic diseases require further study. Third, characterizing potential health disparities is essential to designing and directing relevant interventions. Fourth, studies describing the effect of multiple chronic conditions on health-related quality of life and economic studies concerning the direct and indirect costs exacted on the economy by people with multiple chronic conditions are also useful in gauging the clinical and public health burden of these conditions. Fifth, research is needed to characterize the proportions of patients with multiple chronic conditions who are candidates for nonpharmacological treatments and to define possible contraindications or special considerations for subsets of patients. Clinical research examining optimal therapeutic lifestyle treatment models, including optimal composition of therapeutic lifestyle modification and delivery mode, for patients with different combinations of multiple chronic conditions can provide clinicians with evidence-based approaches to managing such patients. Finally, past studies of people with a predominant condition can be useful to inform the development of a generation of studies focused on people with multiple chronic conditions.

The results of our study suggest that the burden of selected major lifestyle-related chronic conditions is increasing slowly but steadily in the United States, a trend that has serious implications for health care costs and the future delivery of health care in the United States. The recently developed HHS strategic framework with national-level strategies for managing patients with multiple chronic conditions is a timely and prudent coordinated response to an evolving public health challenge (7,8). Continued surveillance of the trend in lifestyle-related chronic conditions with data from the NHIS and other data systems can provide critical feedback to track the evolution of the temporal, spatial, and sociodemographic dimensions of multiple lifestyle-related chronic conditions that will allow timely adjustments to the nation’s health care system to mitigate the effect of this mounting public health concern.

## Appendix. Questions From the National Health Interview Survey^a^ Used to Identify Participants With a Chronic Condition.

Have you ever been told by a doctor or other health professional that you had coronary heart disease?

Have you ever been told by a doctor or other health professional that you had angina, also called angina pectoris?

Have you ever been told by a doctor or other health professional that you had a heart attack (also called myocardial infarction)?

Have you ever been told by a doctor or other health professional that you had a stroke?

Have you ever been told by a doctor or other health professional that you had cancer or a malignancy of any kind?

Have you ever been told by a doctor or other health professional that you had emphysema?

Have you ever been told by a doctor or health professional that you have diabetes or sugar diabetes?

Have you ever been told by a doctor or other health professional that you have some form of arthritis, rheumatoid arthritis, gout, lupus, or fibromyalgia?

During the PAST 12 MONTHS, have you been told by a doctor or other health professional that you had chronic bronchitis?

^a^ National Health Interview Survey (10).
