# Erratum, Vol. 10, April 26 Release

**DOI:** 10.5888/pcd10.120316e

**Published:** 2013-05-09

In the article “Co-Occurrence of Leading Lifestyle-Related Chronic Conditions Among Adults in the United States, 2002-2009,” the wrong label values appeared on the y-axis of the figure because of an editing error. The values should have been stated in tens of millions and instead were stated in millions. The correction was made to our web site on May 1, 2013, and appears online at http://www.cdc.gov/pcd/issues/2013/12_0316.htm. We regret any confusion or inconvenience this error may have caused.

